# Cervical cancer awareness and risk factors among women residing in an urban slum in Lagos, Southwest Nigeria

**DOI:** 10.4314/ahs.v23i3.33

**Published:** 2023-09

**Authors:** Tope Olubodun, Oluwatoyin O Ogundele, Zainab A Salisu, Yetunde O Odusolu, Ugonnaya U Caleb-Ugwuowo

**Affiliations:** 1 Department of Community Health and Primary Care, Lagos University Teaching Hospital, Lagos, Nigeria; 2 Ladiya Hospital, Sabon-gari, Zaria, Kaduna State, Nigeria

**Keywords:** Cervical cancer, awareness, risk factors, slum, Nigeria

## Abstract

**Background:**

Poor awareness of cervical cancer and high prevalence of its risk factors may be responsible for the large burden of cervical cancer in low-income countries. This study assessed awareness of cervical cancer and prevalence of risk factors among women residing in a slum in Lagos, Nigeria.

**Methods:**

This was a descriptive cross-sectional study carried out amongst 305 women of reproductive age (15-49 years) in Idi-Araba, a slum in Urban Lagos. Data were collected using interviewer administered questionnaires. Analysis was done with SPSS 20 software.

**Results:**

Mean age of respondents was 33.5(9.0) years. Only 12.8% of the respondents had heard of cervical cancer. Ninety-five percent of respondents were sexually active and 56.2% had more than one lifetime sexual partner. Close to half (47.3%) of respondents had their first sexual intercourse before the age of 20. One in five (22.2%) had 5 or more children. Half of the respondents (54.8%) had had abnormal vaginal discharge.

**Conclusion:**

Awareness of cervical cancer among the women was poor and prevalence of risk factors of cervical cancer was high. Campaigns aimed at increasing awareness of cervical cancer, and screening should be carried out by governmental and charitable organizations for women residing in slums.

## Introduction

Worldwide, cervical cancer ranked as the 4^th^ most common cancer and cause of cancer related deaths in women with estimated 570,000 new cases and 311,000 deaths in 2018. 1 Cervical cancer has remained a huge public health burden in many low- and middle- income countries worldwide. 1 The highest regional rates and mortality are seen in Africa. 1 In Nigeria, cervical cancer is the commonest gynaecological cancer. An estimated 12,000 new cases of cervical cancer and 8,000 deaths due to the disease were recorded in 2020. 2 Nigeria has an estimated five-year prevalence of 22.11% for cervical cancer as published in GLOBOCAN fact sheets of 2020. 2

Human papillomavirus (HPV) is the most common viral infection of the reproductive tract. 3 Most sexually active women and men will be infected at some point in their lives and some may be repeatedly infected. There are many types of HPV, and many do not cause problems. HPV infections usually clear up without any intervention within a few months after acquisition, and about 90% clear within 2 years. Although most HPV infections clear up on their own and most pre-cancerous lesions resolve spontaneously, there is a risk for all women that HPV infection may become chronic and pre-cancerous lesions progress to invasive cervical cancer. 3

Certain types of sexual behaviours are considered risk factors for cervical cancer and HPV infection. The age at which women engage in their first sexual intercourse has been cited as a risk factor for cervical cancer as damage might be caused to the cervix at a time when it is still developing.^4^ Women who begin sexual activity before age 20 years are at higher risk of cervical cancer.^5^ The greater number of sexual partners a woman has, the greater her risk of coming into contact with HPV and of later developing cervical cancer.^4^ Having sex with someone who has had multiple partners also increases ones risk of coming into contact with HPV and of later developing cervical cancer.^6^

Women who have had 3 or more full-term pregnancies have an increased risk of developing cervical cancer. 6 Studies have pointed to hormonal changes during pregnancy as possibly making women more susceptible to HPV infection or cancer growth. Another thought is that pregnant women might have weaker immune systems, allowing for HPV infection and cancer growth. 7

Age at first full term labor is also an identified risk factor for cervical cancer. Age at first full term labor < 20 years has an increased risk of cervical cancer. 8 Women who smoke are more likely than non-smokers to get cervical cancer. Tobacco by-products have been found in the cervical mucus of women who smoke. These substances damage the DNA of cervix cells and may contribute to the development of cervical cancer. Smoking also makes the immune system less effective in fighting HPV infections. 9

There is evidence that taking oral contraceptives (OCs) for a long time, up to 5 years increases the risk of cancer of the cervix. Research suggests that the risk of cervical cancer goes up the longer a woman takes OCs, but the risk goes back down again after the OCs are stopped, and returns to normal about 10 years after stopping. 7 In most people with healthy immune systems unlike those who are immunosuppressed, the HPV virus clears itself from the body within 12-18 months. 6 The immune system is important in destroying cancer cells and slowing their growth and spread. In women with HIV, a cervical pre-cancer might develop into an invasive cancer faster than it normally would. Another group of women at risk for cervical cancer are those taking immunosuppressive drugs.

It is thought that having a sexually transmitted infection (STI) increases the chance that a woman will also have HPV. Research suggests that long-term inflammation caused by certain STIs may increase the risk of cervical cancer in women with HPV. Studies also show a link between Chlamydia and Herpes infection and cervical cancer. 6 Women of low socio-economic status have a higher risk of developing cervical cancer. This is mainly because these women are less likely to get regular pap tests as they have limited knowledge and access to reproductive health services. 10 Some studies have also shown higher prevalence of HPV DNA among the poor as a result of high rates of risky behaviour. 11 Low awareness and poor knowledge on cervical cancer have been identified as major barriers to effective cervical cancer prevention. 12, 13, 14, 15 Assessing the awareness of cervical cancer among women could be one of the first approaches towards designing a successful cervical cancer prevention programme for them. Identifying the risk factors of cervical cancer among this high-risk group can be instrumental in designing interventions in the control of cervical cancer. This study was carried out to assess the awareness and prevalence of risk factors of cervical cancer among women residing in an urban slum in Lagos, Nigeria.

## Methods

### Study area

Lagos State is the smallest state in Nigeria but Nigeria's economic and commercial capital. 16 Lagos State comprises of 20 local government areas. 16 With an estimated population of 21 million in 2016, Lagos is the most populous state in Nigeria and the largest city in Africa. 17 This study was carried out in Idi-araba, a slum in Lagos, Nigeria. Idi-araba is one of the ten political wards in Mushin Local government area of Lagos. It is a densely populated, overcrowded slum with residential houses and shops. The area is known as a settlement for the Hausa people although the most common tribe is the Yoruba tribe.

### Study design and study population

This study is a descriptive cross-sectional study. Inclusion criteria: women aged 15 – 49 years who had resided in Idi-araba for at least 2 years prior to the study. Exclusion: women who had a diagnosis of cervical cancer.

### Sampling methodology

The sample size was determined using the Cochran formulae: n = Z^2^ pq/ d^2^

With p (19.5%) as the proportion of respondents with adequate knowledge of cervical cancer from a previous study in Ile-Ife, southwest Nigeria, 18, minimum sample size came to 241. Adding a non-response rate of 30%, the sample size came to 344. The questionnaires completely answered were 305 meaning a response rate of 88%.

Respondents were selected using multistage technique. The first stage involved selection of twenty streets out of the total number of streets in Idi-araba (fifty one streets) using simple random sampling. The second stage involved use of systematic sampling to select houses on each of these twenty streets until the desired number of houses was met. Fifteen houses were selected on 15 streets each while on the remaining 5 streets, sixteen houses were selected each to make a total of 305 houses. The sampling interval k for each street was calculated as n/N for each street where n = total number of houses on the street and N = number of houses to be selected. Where there was more than one eligible female in a house, the respondent was selected by balloting.

### Data collection

Data was collected using interviewer administered questionnaire. The questionnaire was adopted from previous studies. 13, 19, 18 The questionnaire contained questions on: socio-demographic characteristics, awareness of cervical cancer, and respondents' risk factors for cervical cancer. The questionnaire was pre-tested among 25 women of reproductive age group in Isolo, Local government Area, Lagos. Two research assistants with a minimum of ordinary level diploma (O.N.D.) qualification were recruited for the study. They received a one-day training and assisted in data collection.

### Data analysis

Data was entered into Microsoft Excel 2010. Analysis was done with SPSS version 20 software. Continuous data was summarized in tables using means and standard deviations. Categorical data was summarized in tables using frequencies and proportions. Univariate logistic regression was done for each of the independent variables and the outcome variable – awareness of cervical cancer. In order to improve the chances of retaining meaningful confounders, variables found significant at p ≤ 0.15 from the univariate logistic regression were included in the multivariate logistic regression. 20 These are: ethnicity, level of education, occupation and religion. Level of significance for multivariable analysis was set at p ≤ 0.05.

### Ethical consideration

Ethical clearance was obtained from the human research and ethics committee of the Lagos University Teaching Hospital. (Approval number: ADM/DCST/HREC/APP/300). Written informed consent was obtained from each respondent and participants were not coerced. Confidentiality was ensured.

## Results

The mean age of respondents was 33.5(9.0) years. Majority (73.1%) of the respondents were married. Of those married, 29.6% were in a polygamous relationship. About half of the respondents were of the Yoruba tribe 166 (54.4%). A higher proportion of respondents had attained secondary education as their highest level of education 165 (54.1%). Majority of the respondents were semi-skilled 216 (70.8%). The respondents that were of the Christian religion were 136 (44.6%) while those of the Islamic religion were 167 (54.8%) (Table 1).

**Table 1:** Socio-demographic characteristics of respondents

Variables	Frequency (n=305)	Percentage
**Age (years)**		
15-19	15	4.9
20-29	99	32.5
30-39	94	30.8
40-49	97	31.8
Mean age =33.5(9.0)		
**Marital Status**		
Single	57	18.7
Married	223	73.1
Divorced/Separated	25	8.2
**Type of Marriage (n=223)**		
Monogamous	157	70.4
Polygamous	68	29.6
**Ethnicity**		
Yoruba	166	54.4
Hausa	60	19.7
Ibo	43	14.1
Others	36	11.8
**Highest Level of Education**		
No formal	38	12.5
Primary	76	24.9
Secondary	165	54.1
Tertiary	26	8.5
**Occupation**		
Unemployed	64	21.0
Unskilled	12	3.9
Semi-skilled	216	70.8
Skilled	13	4.3
**Religion**		
Islam	167	54.7
Christianity	136	44.6
Traditional Religion	2	0.7

Others (minority tribes): Calabar, Cross river, Delta, Edo, Igala, Tapa

Of the respondents that had heard of cancer, 293 (98.7%) knew about breast cancer. Most (87.2%) respondents were not aware of cervical cancer. Of the 39 respondents that had heard of cervical cancer, 20(51.3%) had heard from the media, 9 (23.1%) from the hospital, 6 (15.4%) from religious organizations, 5 (12.8%) from relatives and 2 (5.1%) from the internet (Table 2).

**Table 2:** Respondents awareness about cancer and cervical cancer

Variables	Frequency (n=305)	Percentage
**Ever heard of Cancer**		
Yes	301	98.7
No	4	1.3
Types of cancer known (n=301) *		
Breast cancer	293	98.7
Lung cancer	39	12.8
Prostate cancer	16	5.2
Cervical cancer	39	13.0
Others	3	1.0
**Ever heard of Cervical cancer (n=305)**		
Yes	39	12.8
No	266	87.2
Source(s) of information about Cervical cancer (n=39) *		
Friends / Relatives	5	12.8
Media (Television, Radio, Newspaper, Magazines)	20	51.3
Hospital	9	23.1
Religious organization	6	15.4
Internet	2	5.1
Religious organization	6	15.4

* Multiple response

Most respondents had had sexual intercourse. The mean age of sexual debut was 19.71 years. Of the 292 respondents that had initiated sexual activity, 128 (43.8%) had only one lifetime sexual partner. Almost half (47.3%) of those that had had their first sexual intercourse, had it before 20 years of age. The respondents that had their first childbirth < 20 years were 60 (23.2%). The mean age at first childbirth was 22.90 years. The respondents that had 3-4 children were 115 (44.4%) and those that had >5 children were 17 (5.8%). About half of the respondents 167 (54.8%) have had abnormal vaginal discharge. Only 22(7.2%) of the respondents used combined oral contraceptive pills and no respondent used tobacco (Table 3).

**Table 3:** Risk factors of cervical cancer among respondents

Variables	Frequency	Percentage (%)
**Ever had sex (E=e05)**		
Yes	292	95.7
No	13	4.3
**Number of lifetime sexual partner (n=292)**		
1	128	43.8
2-3	126	43.2
4-5	21	7.2
>5	17	5.8
**Mean( SD) 2.26(1.96)**		
**Age at first sex (n=292)**		
<20	138	47.3
≥ 20	154	52.7
**Mean(SD) 19.8(3.2)**		
**Has at least one childbirth (n=292)**		
Yes	259	88.7
No	33	11.3
**Number of children (n=259)**		
1-2	87	33.6
3-4	115	44.4
≥5	57	22.0
**Mean (SD)** 3.08(1.83)		
**Age at first childbirth (n=259)**		
<20	60	23.2
20 – 29	175	67.6
30 – 39	24	9.2
**Mean(SD) 22.90(4.61)**		
**Ever had abnormal vaginal discharge (n=305) (n=305)**		
Yes	167	54.8
No	138	45.2
**Received treatment for this vaginal Discharge (n =167)**		
Yes	160	95.8
No	7	4.2
**Place received treatment for Abnormal vaginal discharge (n = 167)**		
Health facility/Health worker	120	71.9
Patent medicine store/ chemist	31	18.6
Traditional healer	2	1.2
Self medication	14	8.4
**Use of oral contraceptive pills (n = 305)**		
Yes	22	7.2
No	283	92.8
**Use of Tobacco (n = 305)**		
No	305	100.0

There was a statistically significant relationship between ethnicity and awareness of cervical cancer as higher proportion of respondents from minority tribes (25.0%) were aware of cervical cancer (p = 0.002). There was a statistically significant relationship between respondents' education and awareness of cervical cancer (p <0.001) as more respondents with tertiary education (65.4%) were aware of cervical cancer. There was also a statistically significant relationship between occupation and awareness of cervical cancer (p <0.001), and between religion and awareness of cervical cancer (p = 0.001). More respondents who were skilled (69.2%) and more respondents who were Christians were aware of cervical cancer. (20.6%). There was no statistically significant association between age, marital status and awareness of cervical cancer (Table 4).

**Table 4:** Association between socio -demographic characteristics of respondents and awareness of cervical cancer

Variables	Aware of Cervical Cancer		OR (95% CI)	p-value
	Yes (n = 39)	No (n = 266)		
**Age (years)**				
15-19 (ref)	1(6.7)	14(93.3)	1	
20-29	10(10.1)	89(89.9)	1.57(0.19 – 13.27)	0.667
30-39	13(13.8)	81(86.2)	2.25(0.27 – 18.56)	0.452
40-49	15(15.5)	82(84.5)	2.56(0.31 – 20.96)	0.381

**Marital Status**				
Single (ref)	7(12.3)	50(87.7)	1	
Married/ Cohabiting	29(13.0)	194(87.0)	1.06(0.44 – 2.58)	0.884
Divorced/Separated	3(12.0)	22(88.0)	0.97(0.23 – 4.12)	0.971

**Ethnicity**				
Yoruba/Hausa (ref)	22(9.7)	204(90.3)	1	
Ibo	8(18.6)	35(81.4)	2.12 (0.88 – 5.14)	0.096
Others	9(25.0)	27(75.0)	3.09 (1.29 – 7.40) *	0.011

**Highest level of education**				
No formal (ref)	0(0.0)	38(100.0)	**1**	
Primary	5(6.6)	71(93.4)	1.27(0.23 – 6.86)	0.783
Secondary	17(10.3)	148(89.7)	1.80(0.39 – 8.23)	0.448
Tertiary	17(65.4)	9(34.6)	34.00(6.61 – 174.78)	<0.001

**Occupation**				
Unemployed (ref)	7(10.9)	57(89.1)	1	
Unskilled	0(0.0)	12(100.0)	1.08(0.20 – 5.79)	0.927
Semi-skilled	23(10.6)	193(89.4)	0.82(0.33 – 2.02)	0.659
Skilled	9(69.2)	4(30.8)	4.13(4.13 – 70.28)	<0.001

**Religion**				
Islam (ref)	11(6.6)	156(93.4)	**1**	
Christianity	28(20.6)	108(79.4)	3.51 (1.67 – 7.38) *	0.001
Traditional Religion	0(0.0)	2(100.0)	14.18 (0.83 – 242.36)	0.067

Others (minority tribes): Calabar, Cross river, Delta, Edo, Igala, Tapa

Predictors of awareness of cervical cancer were level of education and religion. Respondents with tertiary education were more likely to be aware of cervical cancer than those with no formal/primary education (AOR 24.30 95%CI 6.27 – 94.14). Christians were more likely to be aware of cervical cancer than respondents of the Islamic/Traditional religion (AOR 3.40 95%CI 1.32 – 8.73) (Table 5).

**Table 5:** Predictors of cervical cancer awareness

	AOR	95% CI	P value
**Ethnicity**			
Yoruba/Hausa (ref)	1		
Ibo	0.92	0.30 – 2.85	0.886
Others	2.12	0.72 – 6.31	0.175

**Highest Level of Education**			
No formal/Primary (ref)	1		
Secondary	2.36	0.81 – 6.84	0.116
Tertiary	24.30	6.27 – 94.14	**<0.001**

**Occupation**			
Unemployed/ Unskilled(ref)	1		
Semi-skilled	0.74	0.27 – 2.03	0.561
Skilled	3.23	0.53 – 19.81	0.206

**Religion**			
Islam/ Traditional religion (ref)	1		
Christianity	3.40	1.32 – 8.73	**0.011**

Others (minority tribes): Calabar, Cross river, Delta, Edo, Igala, Tapa

## Discussion

Most of the respondents had heard about cancer. The majority knew about breast cancer but only a few had heard about cervical cancer. This may be because the breast is an external organ and can be easily recognized unlike the cervix which can only be visualized with the aid of a speculum. Several studies among slum dwellers have shown low awareness of cervical cancer. 13, 21, 12 Awareness of cervical cancer reported in Makoko and Abete slum communities of Lagos was 4.2% of respondents, even lower than our study. 13 A study in the slums of Old Hubli Karnataka, India, showed that only 7.5% of the respondents had heard about cervical cancer. 22 The low level of education of these group of women may account for the poor awareness of cervical cancer.

In a community-based study in Okada, Edo state, southern Nigeria, only 9% were aware of cervical cancer. 23 This is lower than what was reported in our study. The study in Okada was carried out in a rural area and this could be responsible for the low awareness. On the other hand, in a study among mothers, in Shomolu local government area of Lagos state, 79.6% had heard of cervical cancer. 24 More of the respondents in the Shomolu study had tertiary level of education and majority of them where skilled and professionals unlike this study where most were semi-skilled and had secondary level of education. The lower level of education in our study could be responsible for poorer knowledge as low level of education is usually associated with limited access to health information.

In our study education was a predictor of awareness of cervical cancer. Respondents with tertiary education were more likely to be aware of cervical than those with no formal/primary education. Similarly, in a study carried out in a resettlement colony of North West Delhi, the proportion of women who were aware of cancer of the cervix increased as the literacy status increased, and the association was statistically significant. 25 This finding is not surprising, as the higher the level of education of a person, the more likely to come across health-related information, including information on cervical cancer. Health education interventions should especially adopt strategies that engage women with lower levels of education.

Christians were more likely to be aware of cervical cancer than respondents of the Islamic/Traditional religion. A similar finding was seen in a study among women in Taraba, northeast Nigeria. 26 Involving religious institutions in health education will help reach women with information on cervical cancer and screening. This is also an important way to reach women who do not attend schools.

Some studies have shown that sex before age 20 years of age and sex with multiple partners are risk factors for cervical cancer. 5, 6 In the current study, close to half of the respondents had had their first sexual intercourse before 20 years of age, and this poses an increased risk of cervical cancer. Also, more than half of respondents had more than one lifetime sexual partner. In another study in slums in Lagos, 60.0% of the women had had their first sexual intercourse before 20 years. 13 A cross-sectional study among women in Aba, south-eastern Nigeria, reported that 63.5% of respondents had multiple sexual partners. 27 In a study among female university students in South Africa, about half had initiated coitus before 18 years and 73.6% had multiple sexual partners. 28 It is not uncommon to find high risk sexual behaviour among university undergraduates.

Women who have had three or more full-term pregnancies, are twice as likely to get cervical cancer. 6 In this study, most had at least three children, with over twenty percent having at least five children. In a study among women in Abakaliki, southeast Nigeria, there was a high prevalence of high parity with 76.0% having at least five children. 19 Cultural practices in south-eastern Nigeria encourage having many children, hence the finding in the Abakaliki study.

Studies show a link between Human papilloma virus (HPV) infection and cervical cancer. In fact, a history of treatment for abnormal vaginal discharge (an indicator of vaginal or cervical infection) also makes the likelihood of HPV infection more. About half of the respondents in this study had had abnormal vaginal discharge at some point. Of the respondents that had had abnormal vaginal discharge, most (71.9%) had received treatment from a health worker. Receiving treatment for the discharge is an indicator of the severity of the discharge. The symptoms were likely disturbing enough in these people to warrant treatment by a health worker. This is a risk factor for cervical cancer because inflammation produced by STIs may increase the risk of cervical cancer in women with HPV. Also, a history of STI makes infection with HPV more likely. A similar finding had been observed in Abakaliki where more than half of respondents had experienced vaginal discharge and most had sought treatment from hospital and doctors. 19

Primary prevention of cervical cancer is based essentially on healthy lifestyles and vaccination against HPV. Primary prevention includes provision of health information and warnings about tobacco use, sexuality education tailored to age and culture, condom promotion/provision for those engaged in sexual activity and male circumcision. 29 Determining the prevalence of risk factors of cervical cancer among women can be important in designing health education messages that will benefit those women and also younger boys and girls in the community with the aim of reducing the risk of HPV transmission.

## Study limitation

Due to the cross-sectional study design, causal relationships cannot be deduced. Also, the study was carried out in one slum area in Lagos State, South West Nigeria. This could limit the generalizability of the study.

## Conclusion

There was a fairly high prevalence of cervical cancer risk factors including age of sexual debut, number of lifetime sexual partners, number of children, age at first childbirth and type of marriage. Awareness of cervical cancer was poor. Respondents with tertiary education, and Christians were more likely to be aware of cervical cancer. Health education interventions are imperative to increase awareness and also knowledge of cervical cancer among women residing in slums. Interventions should also target women with low level of education and engage religious bodies.

## Role of authors

TO conceptualized the study had the primary responsibility of designing of the study protocol, data collection, data analysis, and writing of the manuscript. OOO, ZAS, YOO, UUC assisted in designing the study protocol, writing and reviewing the manuscript. All authors have read and approved the manuscript.
